# Ferulic acid lowers body weight and visceral fat accumulation via modulation of enzymatic, hormonal and inflammatory changes in a mouse model of high-fat diet-induced obesity

**DOI:** 10.1590/1414-431X20165630

**Published:** 2017-01-05

**Authors:** T.S. de Melo, P.R. Lima, K.M.M.B. Carvalho, T.M. Fontenele, F.R.N. Solon, A.R. Tomé, T.L.G. de Lemos, S.G. da Cruz Fonseca, F.A. Santos, V.S. Rao, M.G.R. de Queiroz

**Affiliations:** 1 Faculdade de Farmácia, Universidade Federal do Ceará, Fortaleza, CE, Brasil; 2 Laboratório de Produtos Naturais, Departamento de Fisiologia e Farmacologia, Faculdade de Medicina, Universidade Federal do Ceará, Fortaleza, CE, Brasil; 3 Faculdade de Medicina Veterinária, Universidade Estadual do Ceará, Fortaleza, CE, Brasil; 4 Departamento de Química Orgânica e Inorgânica, Universidade Federal do Ceará, Fortaleza, CE, Brasil

**Keywords:** Ferulic acid, Anti-obesity, High-fat diet, Leptin, Lipase, Tumor necrosis factor-α

## Abstract

Previous studies have reported on the glucose and lipid-lowering effects of ferulic acid (FA) but its anti-obesity potential has not yet been firmly established. This study investigated the possible anti-obesitogenic effects of FA in mice fed a high-fat diet (HFD) for 15 weeks. To assess the antiobesity potential of FA, 32 male Swiss mice, weighing 20–25 g (n=6–8 per group) were fed a normal diet (ND) or HFD, treated orally or not with either FA (10 mg/kg) or sibutramine (10 mg/kg) for 15 weeks and at the end of this period, the body weights of animals, visceral fat accumulation, plasma levels of glucose and insulin hormone, amylase and lipase activities, the satiety hormones ghrelin and leptin, and tumor necrosis factor-α (TNF-α) and monocyte chemoattractant protein-1 (MCH-1) were analyzed. Results revealed that FA could effectively suppress the HFD-associated increase in visceral fat accumulation, adipocyte size and body weight gain, similar to sibutramine, the positive control. FA also significantly (P<0.05) decreased the HFD-induced elevations in serum lipid profiles, amylase and lipase activities, and the levels of blood glucose and insulin hormone. The markedly elevated leptin and decreased ghrelin levels seen in HFD-fed control mice were significantly (P<0.05) reversed by FA treatment, almost reaching the values seen in ND-fed mice. Furthermore, FA demonstrated significant (P<0.05) inhibition of serum levels of inflammatory mediators TNF-α, and MCH-1. These results suggest that FA could be beneficial in lowering the risk of HFD-induced obesity via modulation of enzymatic, hormonal and inflammatory responses.

## Introduction

The increasing trend in the prevalence of obesity has become a global concern, causing severe burden on health care systems (1). Characterized by the accumulation of excess adipose tissue, obesity is related to cardiovascular disease, insulin resistance, and metabolic syndrome (2). The available anti-obesity drugs such as orlistat and sibutramine (SIB) have modest clinical efficacy, but safety and tolerability concerns may limit their use (3,4). Therefore, there is a need for the discovery and development of novel, safe, and effective drugs for the control and treatment of obesity.

**Figure 1:**
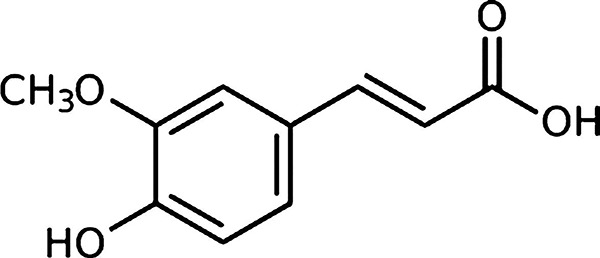
Chemical structure of ferulic acid ((E)-3-(4-hydroxy-3-methoxy-phenyl)prop-2-enoic acid).

Due to the increasing consumer knowledge of the link between diet and health, there is an increased awareness and demand for functional food ingredients and nutraceuticals. This leads to a mindset directed to self-medication to avoid the undesirable side effects associated with consumption of synthesized drugs and also to avoid the increasing cost of drug therapy. Several studies have focused on prevention and treatment of obesity and its associated health risks using plant-derived phenolic compounds. The nutritional effects of phenolic compounds have been well established in their role in modulating specific physiological functions in rodents and human beings (5,6). Ferulic acid (FA) (Figure 1) belongs to the family of phenolic acids and is very abundant in fruits, vegetables and grains, such as rice bran, oats, wheat, barley, roasted coffee, tomatoes, asparagus, berries, vegetables, citrus fruits and leaves of most plants. FA has shown to exhibit hypocholesterolemic, hypoglycemic, anti-atherogenic, anticancer, antioxidant, antidiabetic and anti-inflammatory properties in experimental studies (7–12). Moreover, FA is a phenolic acid of low toxicity and it can be absorbed, easily metabolized in the human body (13). Recently, FA has also shown to improve the glucose and lipid homeostasis in high-fat diet (HFD)-fed mice probably via modulating the expression of lipogenic and gluconeogenic genes in liver tissues (14).

The consumption of fat-rich foods may activate an inflammatory response in the hypothalamus, thereby disturbing the anorexigenic and thermogenic signals generated by the hormones, ghrelin, leptin and insulin, leading to anomalous body mass control (15). Since FA exhibits anti-oxidant (9 [Bibr B10] [Bibr B11]) and anti-inflammatory properties (16), and modulatory effects on glucose and lipid metabolism (14,17), it is likely that FA modulates adipogenesis. Therefore, the present study was aimed to demonstrate the anti-obesity effect of FA (10 mg/kg) compared to sibutramine (10 mg/kg), a well-known weight loss agent, using the mouse model of HFD-induced obesity.

## Material and Methods

### Chemicals and drugs

Ferulic acid was purchased from Sigma Aldrich® (USA), 98% pure. Sibutramine was purchased from Aché® (Brazil). All other chemicals and reagents used were of analytical grade and obtained from standard commercial suppliers.

### Animals

Male Swiss mice (32 total, weighing 20–25 g) obtained from the Central Animal House of Universidade Federal do Ceará were used. They were kept in propylene cages, at a room temperature (24±2°C) on a 12-h light/dark cycle with food (chow) and water provided *ad libitum*. Experimental protocols (#34/2011) were approved by the Universidade Federal do Ceará Institutional Committee on Care and Use of Animals for Experimentation, in accordance with the guidelines of the National Institutes of Health, USA.

### Diet composition

The normal diet (ND) used was the pelleted chow obtained from a commercial source (Nuvilab, Brazil). It consisted of 19.0% protein, 56% carbohydrate, 3.5% lipids, 4.5% cellulose, 5.0% vitamins and minerals, and 12% humidity with a total energy content of 17.03 kJ/g. The standardized HFD used for the study (18) comprised the following hypercaloric constituents: 15 g of laboratory animal chow, 10 g of roasted ground nut, 10 g of milk chocolate, and 5 g of maizen cookies. These ingredients were ground and prepared in the form of pellets that contained, by weight, 20% protein, 48% carbohydrate, 20% lipids, 4% cellulose, 5% vitamins and minerals and 3% humidity. The net energy content of this diet was 21.40 kJ/g. Thus, the HFD, compared with ND, was hypercaloric, and it contained less carbohydrate but more lipids with a net energy difference of 4.37 kJ/g. To avoid auto-oxidation of the fat components, food was stored at approximately 24°C.

### Anti-obesity activity

Mice were randomly divided into four groups (n=8) matched for body weight after 1 week being fed laboratory pellet chow. The control group (ND) continued to be fed laboratory pellet chow *ad libitum*. The remaining mice consumed HFD (HFD control), HFD+FA (0.05% in drinking water, which is equivalent to 10 mg/kg of body weight based on water consumption), or HFD+SIB (0.05% in drinking water, which amounts to 10 mg/kg of body weight) for 15 weeks. FA and SIB concentrations were based on our previous experiments (8,19). FA was suspended initially in 3% (v/v) Tween 80 and then further diluted in water. HFD-fed controls received the same vehicle. As SIB is water-soluble, no vehicle was used. FA- or vehicle-containing water was changed twice a week, and weekly consumption of water (mL/week) was recorded. The body weight of each mouse was measured once a week, the total amount of food consumption was recorded every day for 15 weeks, and weekly consumption of food (g/week) was calculated. At the end of this period, animals were fast for 6 h, blood was taken by venous puncture and then they were killed by cervical dislocation. The plasma was prepared and either used within a few hours or frozen at -70°C until analysis. The liver and visceral adipose tissues (epididymal and parametrial) were dissected, weighed, and expressed in mg/10 g of body weight.

### Biochemical analysis

Plasma amylase and lipase activities were determined by a kinetic method using commercial kits for amylase (Labtest®, Brazil) and lipase (Bioclin®, Brazil). The assays were performed according to the manufacturer’s instructions, and their levels are reported in U/L. Plasma glucose, triglycerides, and total cholesterol were analyzed using commercial kits (Labtest®), and the levels are reported as mg/dL. Plasma alanine aminotransferase (ALT) and aspartate aminotransferase (AST), reported in U/L, were analyzed by a kinetic method using commercial kits (Labtest®). Plasma TNF-α, MCP-1, insulin, leptin, and ghrelin levels were measured by enzyme linked immunosorbent assays (Crystal Chem, USA) performed in duplicate and reported in ng/mL or pg/mL.

### Non-protein sulfhydryls (NP-SH)

NP-SH (non-protein sulfhydrils, GSH) in hepatic tissues were determined by Ellman’s reaction using 5’5’-dithio-bis-2-nitrobenzoic acid (DTNB) (20). Aliquots of 4 mL of the homogenates in ice-cold ethylenediaminetetraacetic acid (EDTA; 0.02 mol/L, pH 8.9) were mixed with 3.2 mL of distilled water and 0.8 mL of 50% trichloroacetic acid (TCA). The tubes were centrifuged at 800 *g* for 15 min at 4°C. The supernatant (2 mL) was mixed with 4 mL Tris buffer (0.4 mol/L, pH 8.9) and 0.1 mL of DTNB (0.01 mol/L). The absorbance was measured within 5 min after addition of DTNB at 412 nm. The absorbance values were extrapolated from a glutathione standard curve and reported as µg/g of hepatic tissue.

### Malondialdehyde assay

The concentration of hepatic lipid peroxidation was determined by estimating malondialdehyde (MDA) using the thiobarbituric acid test (21). The hepatic tissue was homogenized in 0.15 KCl, pH 7.4. The homogenate was maintained in a water bath for 60 min at 37°C. Perchloric acid (35%) was added to the homogenate and centrifuged at 17,500 *g* for 10 min at 4°C. The supernatant was mixed with 1.2% thiobarbituric acid, and the mixture was heated at 98°C for 30 min. After cooling to room temperature, the absorbance was measured at 532 nm. The standard curve was obtained using 1,1,3,3-tetramethoxypropane. The results were reported as nmol of MDA/g hepatic tissue.

### Histological analysis

The epididymal fat pads were excised, fixed in 10% formalin, and processed routinely for paraffin embedding. Tissue sections of 5-µm thick were cut, processed for hematoxylin and eosin (H&E) staining, and examined under a light microscope for histological changes.

### Statistical analyses

The results are reported as means±SE for 8 animals in each group. Data were analyzed by one-way analysis of variance (ANOVA) followed by Student-Newman-Keuls test, using the GraphPad (USA) Prism program (version 5.0). Differences were considered to be significant at P<0.05.

## Results

### Antiobesity effect of ferulic acid and sibutramine in mice fed HFD

**Figure 2:**
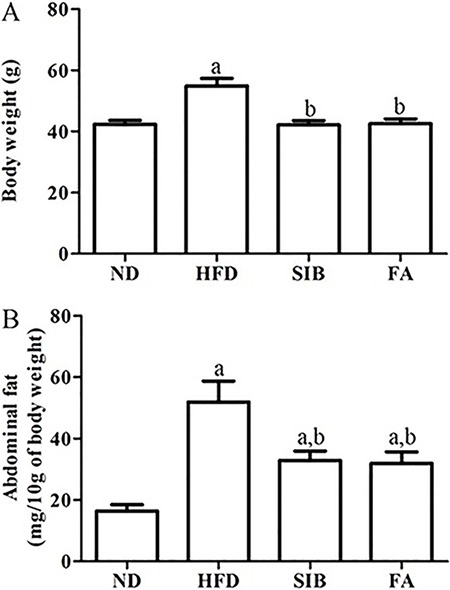
Effect of ferulic acid (FA) and sibutramine (SIB) on body weight (*A*) and abdominal fat content (*B*) in mice fed a HFD during 15 weeks. Results are reported as means±SE. ND: normal diet; HFD: high-fat diet. *a*, P<0.05 *vs* ND; *b*, P<0.05 *vs* HFD (one-way ANOVA followed by Student-Newman-Keuls test).

The body weight of mice in the four groups was similar at baseline and constantly increased over time. As shown in Figure 2A, at the end of the 15-week experimental period, the high calorie diet induced a significant (P<0.05) increase of 29% in body weight of the HFD group compared to the ND group (54.88±2 *vs* 42.36±1.27 g, respectively). Abdominal fat weight, which includes the epididymal and parametrial pads, was significantly (P<0.05) higher in HFD controls compared to ND controls (Figure 2B). FA supplementation in drinking water (50 mg/L) significantly (P<0.05) reduced the final body weight (g) and abdominal fat deposition (mg/10 g) to 42.56±1.54 and 31.95±3.73, respectively, when compared to the HFD group, representing reductions of 22 and 38% in these parameters. The FA had an effect similar to sibutramine in reducing body weight and abdominal fat accumulation (23 and 36%, respectively). While treatment with FA and SIB slightly but significantly (P<0.05) reduced water consumption compared to ND and HFD controls, both treatments effectively decreased the net energy intake almost to the level seen in ND fed mice when compared to values observed in HFD controls (Table 1).

**Table:**
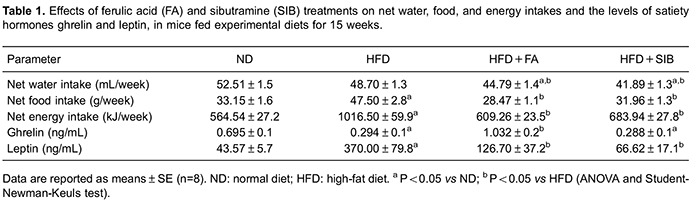

### Effects of ferulic acid and sibutramine on plasma parameters

As shown in Table 2, plasma levels of glucose and insulin were significantly (P<0.05) higher (78% and 3.2-fold, respectively) in the HFD group compared to ND group. FA treatment for 15 weeks led to a significant (P<0.05) 33% reduction in plasma glucose levels and 58% reduction in plasma insulin levels compared to HFD group. Treatment with SIB showed no significant influence on plasma glucose, but it effectively decreased insulin level (48%). Compared to the ND group, the HFD group significantly (P<0.05) decreased plasma ghrelin (orexigenic hormone), while it elevated the leptin (anorexigenic hormone) level (Table 1). These changes induced by the HFD were effectively reversed by FA treatment. SIB treatment, however, caused no significant change in the level of ghrelin hormone.

**Table:**
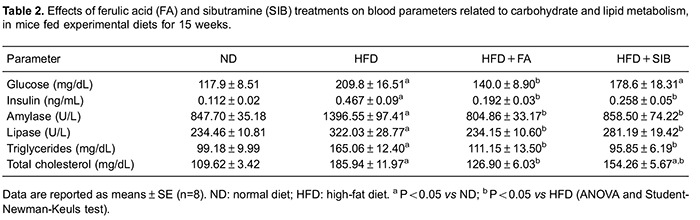

The amylase and lipase activities were significantly (P<0.05) higher in the HFD group (65 and 37%, respectively), compared to ND group. FA treatment caused significant (P<0.05) reductions in the activities of both amylase and lipase by 42 and 27%, respectively. SIB, used as a reference standard, reduced significantly (P<0.05) the amylase activity (38%), while it had no influence on lipase activity (Table 2).

The HFD raised total cholesterol (70%) and triglycerides (66%) significantly (P<0.05) compared with the ND. These increased levels of the HFD group were significantly (P<0.05) lowered by FA and SIB treatments (Table 2).

### Effects of ferulic acid and sibutramine on plasma levels of inflammation and lipid peroxidation-related parameters

Table 3 shows the effects of FA and SIB treatments on plasma levels of monocyte chemotactic protein 1 (MCP-1) and tumor necrosis factor (TNF-α), and hepatic levels of NP-SH, and MDA, in mice fed experimental diets for 15 weeks. The levels of MCP-1 showed an increase of 55% in the HFD relative to ND group, which was significantly (P<0.05) decreased in groups treated with FA and SIB (44 and 34%, respectively). Similarly, the increase in TNF-α promoted by the HFD was also greatly decreased in groups treated with FA and SIB. The hepatic tissue levels of NP-SH, and MDA were significantly (P<0.05) elevated by the HFD, whereas in mice treated with FA or SIB these changes were partially reversed, but not significantly.

**Table:**
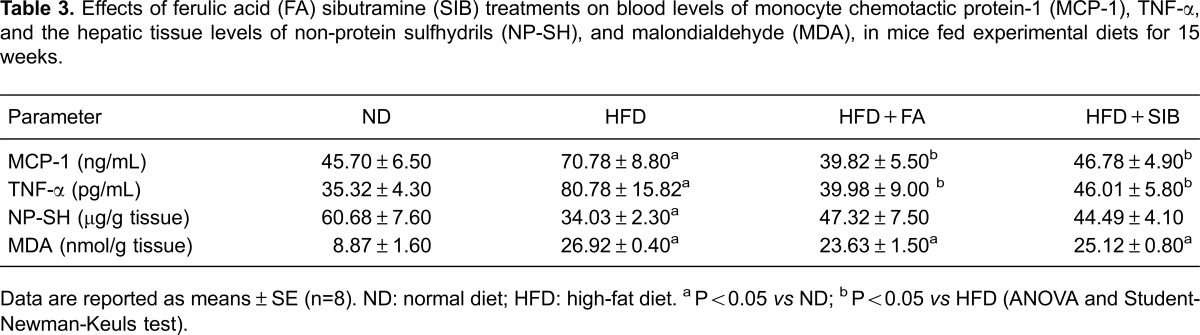

### Effects of ferulic acid and sibutramine on satiety parameters

There were no significant changes in serum urea and creatinine levels among all tested groups. However, animals subjected to the hypercaloric diet showed an increase in the activity of ALT and AST (41 and 46%, respectively), indicating a possible liver damage (Table 4). Serum ALT and AST decreased significantly (P<0.05) in groups treated with FA compared to HFD control group, representing a decrease of 33 and 74% in the activity of the respective enzymes. Sibutramine, used as positive control, also significantly (P<0.05) reduced the activity of ALT and AST by 36 to 59% compared to HFD fed animals.

**Table:**
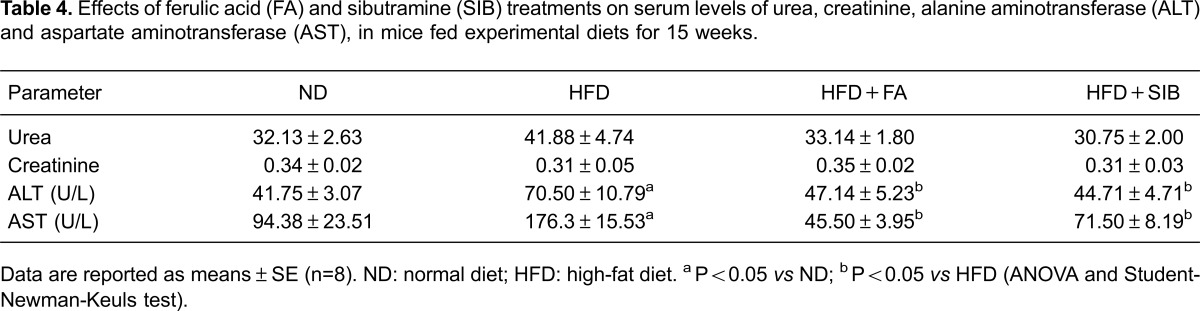

### Effects of ferulic acid and sibutramine on epididymal adipocytes size in mice fed HFD

**Figure 3:**
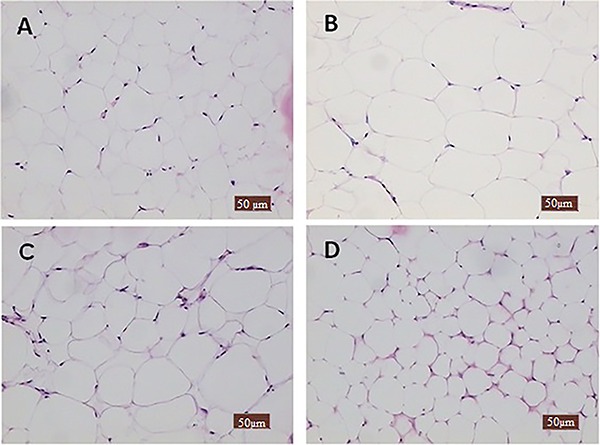
Histology of adipose tissue of mice fed the experimental diets for 15 weeks. Representative microphotographs of mouse epididymal fat pad (*A*), normal diet showing normal architecture of adipocytes (*B*), high-fat diet showing an increased size adipocyte (*C*), high-fat diet+ferulic acid treatment, and (*D*), high-fat diet+sibutramine treatment, which presents smaller adipocytes compared to normal diet-fed animals (H&E, ×100).

Mice fed HFD for 15 weeks apparently presented greater number of epididymal fat cells with an increased size of adipocyte (Figure 3) compared to the group that received normal diet. In the groups treated with FA or SIB, the adipocyte size almost resembled those of the ND-fed mice.

## Discussion

Past studies have addressed the hypoglycemic, hypolipidemic, and antioxidant properties of FA in HFD fed animals (8,9), but its influence in lowering the visceral adiposity has not been analyzed and reported. The results obtained in this study clearly showed that a HFD for 15 weeks promoted visceral adiposity and weight gain in Swiss mice, and treatments with the FA, or a known anorectic agent, such as SIB (22) in drinking water (50 mg/L) prevented this adiposity and weight gain.

Ghrelin from the stomach and leptin from adipose tissue regulate appetite and energy homeostasis in humans and rodents. It has been observed that the long-term intake of a HFD can induce hyperleptinemia and hypoghrelinemia and a significant positive correlation between plasma leptin levels and epididymal fat mass (23). Consistent with these earlier findings, the present study with mice on HFD for 15 weeks demonstrated hyperleptinemia and hypoghrelinemia and an increased abdominal fat deposition, which could be counteracted by FA supplementation. The hypothalamus is considered the major site of anorexigenic and orexigenic signaling integration through activation of respective receptors for ghrelin (orexigenic) and leptin (anorexigenic). Obesity is associated with elevated leptin and resistance to leptin effects on energy homeostasis (24). Our current study demonstrated that FA supplementation significantly lowers circulating leptin level. Since FA supplementation could reduce the food and energy intakes, we assume that the HFD-induced changes in ghrelin and leptin plasma levels may, in part, account for the observed body weight loss and reduced abdominal fat. However, the underlying molecular mechanism of FA in its antiobese effect remains to be established.

Several studies demonstrated that adipose tissue dysregulation and aberrant adipokine secretion contribute to low-grade chronic proinflammatory state and insulin resistance (25–27). Obesity has been considered a disease in which there is a predominance of proinflammatory cytokines. Recent findings suggest that the size of adipocytes is a major modulator of their endocrine function. Hypertrophic adipocytes secrete greater amounts of TNF-α and MCH-1 than normal adipocytes, and this excess secretion has been hypothesized to cause insulin resistance (28–30). In our studies, circulating levels of MCP-1 and TNF-α, as well as insulin were greatly elevated in mice fed on HFD for 15 weeks, indicating a proinflammatory state and insulin resistance seen in type-2 diabetes. Both FA and SIB reduced the adipocyte size, as well as the circulating levels of adipokines, TNF-α and MCH-1, which would explain its ameliorating effect on abdominal adiposity and insulin resistance.

In addition, there was a significant improvement in biochemical parameters such as plasma glucose, cholesterol and triglycerides, as well as the lipase and amylase activities, when mice were treated with the combination of FA plus HFD. Inhibition of pancreatic lipase and the associated reduction of lipid absorption is an attractive approach for the discovery of potent agents (31). Currently, the only clinically approved pharmacological agent for pancreatic lipase inhibitor is orlistat. However, its usage is compromised by unpleasant gastrointestinal adverse reactions (oily stools and flatulence). An important target for the treatment of obesity includes the development of inhibitors of nutrient digestion and absorption. FA is therefore capable of modulating glucose and lipid metabolism, consistent with earlier reports (8,16,32).

Overweight and obesity have a major impact on global health. Strategies for weight control management affect gut hormones as potential targets for the appetite metabolic regulation and stimulation of energy expenditure. Past and current weight-loss medications have serious safety risks. For example, orlistat has a good safety profile but a high rate of gastrointestinal side effects (4). Another example is *Garcinia Cambogia* that although it protects against HFD-induced obesity by modulating adipose fatty acid synthesis and β-oxidation, it can induce hepatic fibrosis, inflammation and oxidative stress (33). In this study, the antiobesity potential of FA is almost comparable to SIB, a weight loss promoting agent that was banned in several countries due to cardiovascular toxicity. However, studies are still relevant as it is often a hidden ingredient in herbal and over the counter slimming products (34). In this context, there are reports that FA may have health benefits by minimizing the cardiovascular complications of metabolic syndrome (35). Besides the antiobese potential of FA, this study also evaluated its likely toxicity to liver and kidney. Elevated serum ALT and AST activities are biomarkers of liver injury (36). ALT and AST were both found to be raised significantly in mice fed the HFD alone compared with ND controls, while FA inhibited this HFD-induced increase. Further, FA did not alter circulating rates of urea and creatinine, which confirms no potential kidney or hepatic damage. Moreover, it prevented HFD-induced fatty liver in the present study and offered protection against diosbulbin-induced hepatotoxicity (37) and glycerol-induced nephrotoxicity (38) in other studies.

This study has its strength in the evaluation of various parameters that are closely related to metabolic syndrome and obesity, which were improved with FA treatment. Adipose tissue and liver are the two main organs involved in lipid metabolism, and transcriptional control of gene expression is a common mechanism by which lipids and other nutrients affect metabolism. The limitation of this study is the absence of analysis of possible genetic changes related to obesity promoted by FA treatment. Therefore, further studies are required to elucidate the antiobesity FA action.

In summary, ferulic acid effectively prevented high fat diet-induced visceral adiposity and body weight gain via mechanisms involving the modulation of food regulatory peptide hormones (insulin, ghrelin and leptin), inhibition of serum amylase and lipase activity, and suppression of adipocyte-derived pro-inflammatory cytokines MCP-1 and TNF-α. These effects of ferulic acid may be beneficial and therefore it might be a promising adjuvant therapy for the treatment of obesity and its complications.
